# What does not kill you makes you Weaker!

**DOI:** 10.1038/s41418-025-01658-6

**Published:** 2026-01-05

**Authors:** Francesca Maremonti, Andreas Linkermann

**Affiliations:** 1 Department of Medicine V, University Medical Centre Mannheim, University of Heidelberg, Mannheim, Germany; 2 Department of Internal Medicine 3, University Hospital Carl Gustav Carus at the Technische Universität Dresden, Dresden, Germany; 3 Division of Nephrology, Department of Medicine, Albert Einstein College of Medicine, Bronx, NY USA

**Keywords:** Tumour heterogeneity, Tumour heterogeneity

## Abstract

Tumor cell evasion from regulated cell death is a hallmark of cancer [1]. Davern et al. now identify resistance to mitochondrial apoptosis to represent the common mechanism by which persister cells that survived either immunotherapy- or irradiation-treatment rely on to proliferate. Therefore, they discovered the persister cells´ Achilles heel for future treatments with BH3-mimetics [2].

The pool of persister cells that survive drug treatment (Drug Tolerant Persister - DTPs) or immunotherapy (Immunotherapy Persister Cells- IPCs) may constitute a reservoir for relapsing cancers that ultimately are resistant to drugs or immunotherapy. But are DTPs resistant to immunotherapy? Are IPCs resistant to conventional chemotherapy? To model persister populations, researchers in the Letai laboratory exposed HeLa cells to high-dose chemotherapy with 5-fluoruracil (5-FU), oxaliplatin, and etoposide at previously identified IC90 doses (a dose that kills 90% of cells within 72 h), generating DTP. In parallel, they established IPCs by co-culturing HeLa and U251 cell lines, following treatment with EGFR-CAR T cells.

A broad set of functional assays was used to probe the biology of these survivor cells. Cell viability and cytotoxicity were assessed with the commonly used CellTiter-Glo® Luminescent Cell Viability assay, the membrane-integrity CellTox assay, and T cell cytotoxicity assays. To specifically examine apoptotic competence, the authors carried out BH3 profiling, both in microscopy- and flow cytometry-based formats, including dynamic BH3 profiling (DBP), a method previously developed in that laboratory [3]. Likewise, FACS-based BH3 profiling was carried out in 384-well plates following stimulation with recombinant BIM peptide and read out in the presence of a cytochrome c antibody. The number of cells that lost cytochrome c within 1 h following incubation with the BIM peptide was interpreted as indicative of the fraction of cells that exhibit particular sensitivity to mitochondrial apoptosis. Through these approaches, the study uncovered that tumor cells that persist following CAR T cell attack are not only less sensitive to several drug types but also acquire cross-resistance to radiotherapy. Similarly, tumor cells that persist following drug therapy (DTPs) were found to obtain cross-resistance to several drug types, T cell killing, and even radiotherapy.

Mechanistically, the reduction of sensitivity to cell death in IPCs was explained by the reduced fraction that was found positive in mitochondrial apoptosis assays, ensured by increased reliance on anti-apoptotic BCL-2 family proteins. Although other cell death modalities, such as necroptosis and ferroptosis, were not explored, the authors demonstrated how this apoptotic vulnerability could be exploited. They identified an apoptotic priming signature, which they found to be specifically altered, a finding that led them to target mitochondrial apoptotic priming. Treatment with BH3 mimetics such as venetoclax successfully re-sensitized persisters, lowering their survival and restoring therapeutic efficacy. This strategy was described as not being limited to IPCs, as targeting these anti-apoptotic dependencies in DTPs reduced their survival in a similar manner and increased sensitivity. Further, comparing DTPs and IPCs, the authors found that both types of persisters shared comparable transcriptional profiles regardless of whether they survived chemotherapy or CAR T cell therapy.

As with every scientific masterpiece, there are more questions following novel observations. How widespread is this mechanism in other cancers? How does it integrate into other mechanisms of persister cells, which may evade conventional chemotherapy by evading apoptosis-independent regulated cell death, such as ferroptosis [4]. Indeed, several manuscripts indicated a major role for metabolic changes, which may submerge into ferroptosis sensitivity following chemotherapy using lung cancer cells made resistant to the tyrosine-kinase inhibitor lapatinib [4, 5]. Indeed, ferroptosis may be regulated by cytochrome c-mediated minority MOMP [6]. To what extent does BH3-mimetic-mediated cell death overlap with such pathways, or are they entirely independent (Fig. 1)? Of course, BH3-mimetics result in a breakdown of the MOMP as well, and they will inevitably be associated with the release of cytochrome c. But those ferroptosis-based strategies to induce persister cell death have a major disadvantage: they are likely to result in untoward effects of ferroptosis-sensitive tissues, which are mostly endocrine organs, and in particular hormone-producing cells, such as pancreatic beta cells and adrenal cells [7], and most prominently the kidney tubular compartment [8]. Ferroptosis-induction may be unsuccessful in premenopausal females, as oestrogens protect from ferroptosis in multiple ways [9]. However, these limitations do not appear to be of therapy-precluding relevance in an apoptosis-inducing approach as followed here by the Letai team. Expected side effects of BH3 may be comparably much less dramatic, and are well understood from phase II [10] and even phase III combination therapy clinical trials for the treatment of acute myeloid leukemia [11]. In that combination trial with azacitidine (a drug for demethylation therapy), the incidence of febrile neutropenia was indeed higher in the combined treatment group. But the overall survival of the patients was prolonged, and the incidence of remission was higher [11]. As a patient, you would want the latter two, and you would accept a chance of fever.

**Fig. 1:**
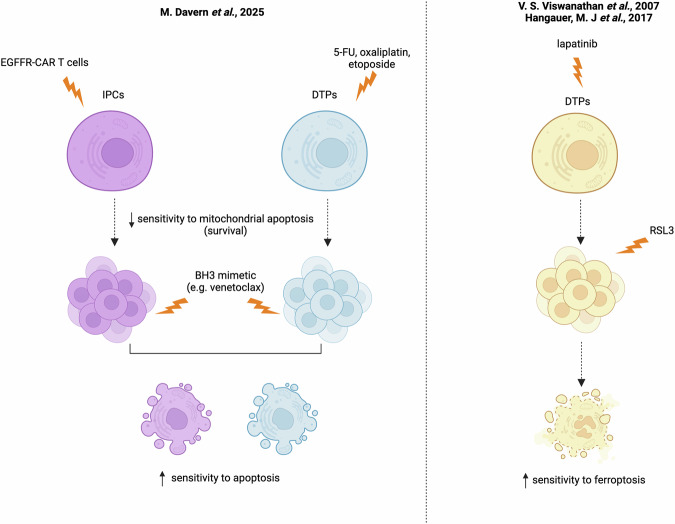
Persister cells evade mitochondrial apoptosis and ferroptosis. Persister cells following immunotherapy (IPCs) or chemotherapeutic drug treatment (DTPs) to be less sensitive to mitochondrial apoptosis. BH3 mimetics can overcome this resistance. These persister cells were induced by 5-fluoruracil (5-FU), oxaliplatin, and etoposide for DTPs and EGFR-CAR T cells for IPCs, respectively. In contrast, investigations of ferroptosis-sensitive DTPs were generated by forced resistance to other drugs, such as the tyrosine-kinase inhibitor lapatinib. In both cases, however, persistence results in an acquired susceptibility to the induction of regulated cell death, be it BH3-induced apoptosis or RSL3-induced ferroptosis. The potential side effect profiles must be prominently discussed if cell death induction therapy is considered for patients. As it stands, venetoclax (a BH3 mimetic) has already entered the clinical routine, while no ferroptosis-inducing drug is currently available.

In summary, there are major differences to be expected if persisters following conventional chemotherapy are compared to persister cells that survived immunotherapy or irradiation. But thanks to this work, the treatment-induced pressure on the cancer cells enforces overarching evasion strategies, the number of which may be limited. Combined cell death induction protocols will provide a crucial contribution to our understanding of such strategies. If the tumor evades one type of cell death, it might become even more sensitive to another one. Along those lines, what does not kill you makes you weaker!
